# Application of Nordic Keyhole and Nutri-Score for assessment of nutritional quality of plant-based dairy analogues

**DOI:** 10.1186/s40795-025-01023-3

**Published:** 2025-02-28

**Authors:** Hanieh Moshtaghian, Elinor Hallström, Marta Bianchi, Susanne Bryngelsson

**Affiliations:** 1 Department of Agriculture and Food, Research Institutes of Sweden (RISE), Gothenburg, Sweden; 2 Department of Agriculture and Food, Research Institutes of Sweden (RISE), Lund, Sweden

**Keywords:** Keyhole, Nutri-Score, Front Of Pack nutrition labelling, Food labelling, Dairy substitutes, Plant Based food

## Abstract

**Background:**

Public interest in plant-based dairy analogues is increasing; thus, their assessment by front-of-pack nutrition labelling schemes such as Keyhole and Nutri-Score can facilitate the identification of products with optimal nutritional quality. In this study, Keyhole and the latest version of Nutri-Score criteria were applied to plant-based dairy analogues (i.e., milk, yoghurt, cheese, cream, fat spread, and ice cream analogues) in the Swedish market to evaluate their nutritional quality.

**Methods:**

Nutritional data for 222 plant-based dairy analogues were collected from food manufacturers’ websites, and the eligibility of these analogues for Keyhole and Nutri-Score (A to E) were assessed. Products eligible for Keyhole and Nutri-Score A or B were deemed to have optimal nutritional quality.

**Results:**

16% of plant-based milk analogues (from oat-, almond-, rice-, and potato-based products), 2% of plant-based yoghurt analogues and 37% of plant-based fat spread analogues were eligible for Keyhole. The plant-based cheese, cream and ice cream analogues were ineligible for Keyhole. None of the plant-based milk analogues qualified for Nutri-Score A, and 45% (mainly soy-, almond-, coconut-, pea- and mixed-based products) qualified for Nutri-Score B. 68% of plant-based yoghurt analogues (from oat-, soy-, almond- and mixed-based products) qualified for Nutri-Score A or B. The plant-based cheese, fat spread and ice cream analogues were ineligible for Nutri-Score A or B and 32% of plant-based cream analogues qualified for Nutri-Score B. A higher percentage of organic milk analogues and a lower percentage of organic yoghurt analogues were eligible for Keyhole and Nutri-Score A or B compared to their non-organic varieties. Keyhole and Nutri-Score had an agreement on classifying two plant-based dairy analogues as optimal nutritional quality products and 133 plant-based dairy analogues as suboptimal.

**Conclusions:**

There is variability in the eligibility of plant-based dairy analogues for Keyhole and Nutri-Score labelling. Eligibility for Keyhole was highest among plant-based fat spread analogues, while Nutri-Score A and B ratings were more common for plant-based yoghurt analogues. Plant-based cheese and ice cream analogues were ineligible for Keyhole and Nutri-Score A or B. Since the micronutrient content of organic and non-organic plant-based dairy analogues did not affect their evaluation by Keyhole and Nutri-Score, this limitation warrants further consideration.

**Supplementary Information:**

The online version contains supplementary material available at 10.1186/s40795-025-01023-3.

## Background

Encouraging people to choose healthy food and drinks is one of the principles of public health nutrition. Authorities have used various strategies, such as proposing food labelling to communicate the nutritional quality of food products to the general public. Front-of-pack Nutrition Labelling (FOPNL) schemes, such as Keyhole [1], Nutri-Score [2], Health Star Rating [3], Healthier Choice Symbol [4] and black-and-white stop sign [5], have been introduced as a tool to guide consumers in selecting healthy foods.

In Europe, the two main voluntary FOPNL schemes are Keyhole and Nutri-Score. The Keyhole has been developed based on Nordic Nutrition Recommendations and is owned by the Swedish Food Agency [1, 6]. It is currently used in Sweden, Norway, Denmark, Iceland, Lithuania and North Macedonia [7]. Keyhole, as a FOPNL scheme, aims to help consumers identify products containing less or healthier fat, less sugar and salt, and more fibre and whole grain compared to similar options in the same food category [1]. The Keyhole eligibility criteria for each food group are based on threshold values for the selected nutrients and ingredients [1]. Keyhole only applies to specific products and food groups, and certain foods (e.g. cakes and biscuits) are considered nonassessable for Keyhole eligibility.

Nutri-Score is a five-letter coloured FOPNL scheme based on the nutrient profiling system preliminary developed by the British Food Standards Agency [8]. It is owned by the Public Health Agency of France [6] and is currently used in France, Belgium, Switzerland, Germany, Spain, the Netherlands and Luxembourg [2]. The Nutri-Score uses specific algorithms for general foods and a few food categories, i.e. beverages, cheese, red meat and fats, oils, nuts and seeds. The Nutri-Score is calculated by considering unfavourable nutritional components (e.g., energy, saturated fat, sugars and salt) to be limited and favourable nutritional components (e.g., fibres, protein, fruits, vegetables, and legumes) to be encouraged [2]. The final score is classified into five categories and displayed in alphabetical order from A (dark green) to E (dark orange), where the Nutri-Score A and E indicate the highest and lowest nutritional quality, respectively. This FOPNL scheme is designed to compare the nutritional quality of foods within the same category rather than across different categories [9, 10].

The FOPNL schemes assess the nutritional quality of both traditional and innovative foods, but some of them, such as Nutri-Score, do not have a specific formula or criteria to differentiate between these types of foods. The plant-based dairy analogues, i.e. plant-based foods designed to mimic dairy, are one of the innovative food groups developed to be consumed as dairy alternatives by people who avoid dairy for medical reasons such as lactose intolerance and allergy or for lifestyle choices, including veganism, environmentalism and animal ethics concern [11–13]. Interest in plant-based dairy analogues has increased in recent years, and the market is estimated to grow in the future. The volume of the milk analogue market in Sweden is expected to increase from 34 million kg in 2023 to 61 million kg in 2028 [14]. The increase in consumption highlights the importance of assessing the nutritional quality of these analogues and the role of FOPNL schemes such as Keyhole and Nutri-Score in guiding consumers.

Most studies in this context compared the nutrient content of plant-based dairy analogues and dairy products to investigate their nutritional quality [15–18], but there are a few studies that assessed the overall nutritional quality of some plant-based dairy analogues based on FOPNL schemes, particularly Nutri-Score [19–23]. In the Nutri-Score studies, certain plant-based dairy products, such as milk and yoghurt analogues, were assessed as optimal nutritional quality choices [19, 21, 22], and cheese analogues were evaluated as suboptimal nutritional quality options [19, 20, 22]. These studies did not include all plant-based dairy analogues such as cream, fat spreads and ice cream analogues.

The findings of the mentioned studies on Nutri-Score assessments may not be valid anymore, particularly after the recent update of the Nutri-Score algorithm in 2023, which included revising the threshold values for points allocated to some components [24] and reclassifying the plant-based milk analogues into the beverage group [25]. Studies on the application of the latest updated version of the Nutri-Score algorithm and also Keyhole are scarce and only focused on a small number of food items in the Food Composition Databases [6, 9].

Since limited studies examined the Keyhole eligibility of plant-based dairy analogues and most of the mentioned studies assessed these analogues based on the previous version of the Nutri-Score algorithm, it is crucial to explore the nutritional quality of all types of plant-based dairy analogues, according to these two FOPNL schemes. Furthermore, as none of the studies focused on the nutritional quality of these products in the Swedish market, the Swedish perspective warrants further investigation. Hence, in this study, Keyhole and the latest version of Nutri-Score criteria were applied to plant-based dairy analogues (i.e., milk, yoghurt, cheese, cream, fat spread, and ice cream analogues) in the Swedish market to evaluate their nutritional quality.

## Methods

### Data collection and food grouping

For the purpose of this study, plant-based dairy analogues were defined as products that imitate dairy and do not contain milk ingredients, e.g., casein, caseinates, whey proteins, and milk fat (butter and anhydrous milk fat). Nutritional data were collected from 222 plant-based dairy analogues (milk, yoghurt, cheese, cream, fat spread and ice cream analogues) available in the Swedish market during 2022. Details of data collection and food grouping were described elsewhere [18]. Briefly, the manufacturers were identified through two popular Swedish supermarket websites (ICA and Coop). Then, product information, including nutrients mandatory and voluntary to declare, ingredient lists and health and nutrition claims, were collected from the manufacturers’ websites for all plant-based dairy analogue groups [18].

Based on the manufacturer’s declaration on the package or webpage, plant-based dairy analogues were grouped into plant-based milk (oat-, soy-, almond-, coconut-, rice-, pea-, potato-, cashew nut-, broad bean- and mixed-based), yoghurt (oat-, soy-, almond-, coconut- and mixed-based), cheese (plant fat-based and plant fat- and protein-based), cream (plant fat-based and plant fat- and protein-based), fat spread, and ice cream analogues [18]. Due to ingredient diversity, plant-based fat spread and ice cream analogues were not divided into subgroups. The classifications of plant-based milk and yoghurt analogues into organic and non-organic varieties were also based on manufacturers’ declarations. It is worth noting that according to the Swedish Food Agency, organic plant-based milk and yoghurt analogues can only be fortified with vitamin D [26].

### Nordic Keyhole assessment

The Keyhole eligibility was evaluated based on adherence to criteria for plain plant-based milk analogues, plain and flavoured plant-based yoghurt, cheese and cream analogues, and plant-based margarine (solid and liquid). Products that met these criteria were Keyhole eligible and deemed to have optimal nutritional quality, and those that did not meet the criteria were Keyhole ineligible and presumed to have suboptimal nutritional quality. In Keyhole regulation, flavoured plant-based milk analogues, plant-based cream cheese, fat spread containing plant sterols and ice cream analogues were not considered for Keyhole eligibility assessment. Therefore, these nonassessable products were also counted towards Keyhole ineligible analogues for this study. The nutritional quality of plant-based milk, yoghurt and cream analogues was examined according to specified threshold values for total fat, saturated fat to total fat ratio, total sugars and salt content (Appendix A). Similar threshold values for these nutrients (except total sugars) were considered to assess the plant-based cheese and fat spread analogues. It should be mentioned that there was no threshold value for the total fat content of liquid fat spread analogues.

### Nutri-Score assessment

Plant-based dairy analogues’ eligibility for Nutri-Score A to E was calculated based on the last update of the Nutri-Score algorithms using the Excel sheet provided by the Public Health Agency of France [2]. All plant-based dairy analogues were assessed for the Nutri-Score. The products scored as A or B were deemed to have optimal nutritional quality, and those qualified for Nutri-Score C, D or E were considered to have suboptimal nutritional quality. Plant-based milk analogues were evaluated based on the Nutri-Score algorithm for beverages, and plant-based yoghurt, cheese and ice cream analogues were assessed based on the Nutri-Score algorithm for general foods. It is worth stating that according to Nutri-Score regulations, plant-based cheese analogues should not be evaluated against cheese criteria [2]. The Nutri-Score algorithm for fats, oils, nuts, and seeds was applied to plant-based cream and fat spread analogues.

For calculating the Nutri-Score of plant-based milk analogues according to the beverage algorithm, points were assigned to components considered unfavourable (energy, saturated fat, sugars, salt and non-nutritive sweeteners) and favourable (protein, fibre, fruit, vegetable and legumes). The same unfavourable (excluding non-nutritive sweeteners) and favourable components were used for Nutri-Score calculation of other plant-based dairy analogues except for cream and fat spread analogues. Unfavourable components for Nutri-Score calculation in plant-based cream and fat spread analogues were energy from saturated fat, saturated fat to total fat ratio, sugars and salt, and favourable components were fruit, vegetable, legumes and oils derived from them, protein and fibre. Similar to other studies [19, 20], the fibre content was assumed to be zero when not declared. The final score was calculated by subtracting favourable points from unfavourable points. The final score was classified into five categories from A to E, according to Appendix B. It is worth mentioning that according to the Nutri-Score algorithm for beverages, none of the plant-based milk analogues qualified for Nutri-Score A, as this Nutri-Score category can only be assigned to water.

### Statistical analyses

The descriptive analysis was conducted to calculate the frequency and percentage of plant-based dairy analogues meeting Keyhole eligibility criteria and qualifying for Nutri-Score A to E. Cross-tabulation was used to assess the frequency and percentage of products classified as optimal or suboptimal nutritional quality by both FOPNL schemes. The agreement between these two schemes for classifying the nutritional quality of products was defined as eligibility for Keyhole and Nutri-Score A or B (agreement in optimal nutritional quality) or ineligibility for Keyhole and qualifying for Nutri-Score C, D or E (agreement in suboptimal nutritional quality). In addition, Kappa statistics were used to evaluate the degree of overall agreement between these two FOPNL schemes in classifying the nutritional quality of plant-based dairy analogues. The Kappa value of less than 0 indicates poor agreement, 0.00 to 0.20 slight agreement, 0.21 to 0.40 fair agreement, 0.41 to 0.60 moderate agreement, 0.61 to 0.80 substantial agreement, and 0.81 to 1.00 almost perfect agreement [27]. All analyses were conducted in SPSS (Version 25, SPSS Inc., Chicago, IL).

## Results

Keyhole eligibility and Nutri-Score classification for each plant-based dairy analogue group are presented in Table 1 and Appendix C. In the plant-based milk analogues, 16% of products were eligible for Keyhole, but none qualified for Nutri-Score A, and 45% qualified for Nutri-Score B. These FOPNL schemes agreed on classifying only one plant-based milk analogue as having optimal nutritional quality. While only 2% of plant-based yoghurt analogues were eligible for Keyhole, more than 68% qualified for Nutri-Score A or B. Only one plant-based yoghurt analogue was classified as an optimal nutritional quality product by both FOPNL schemes. The agreement between Keyhole and Nutri-Score for assessing plant-based cheese analogues as suboptimal nutritional quality products was 100% because none of these products were eligible for Keyhole or Nutri-Score A or B, but the majority of them qualified for Nutri-Score E.

**Table 1:** Nutritional quality of plant-based dairy analogues based on Keyhole and Nutri-Score

	**n**	**Keyhole** **n (%)**	**Nutri-Score** **n (%)**					**Agreement in optimal nutritional quality**^a^ **n (%)**	**Agreement in suboptimal nutritional quality**^b^ **n (%)**
			**A**	**B**	**C**	**D**	**E**		
Plant-based milk analogues	73	12 (16.4)	0 (0.0)	33 (45.2)	24 (32.9)	8 (11.0)	8 (11.0)	1 (1.4)	29 (39.7)
Plant-based yoghurt analogues	41	1 (2.4)	9 (22.0)	19 (46.3)	13 (31.7)	0 (0.0)	0 (0.0)	1 (2.4)	13 (31.7)
Plant-based cheese analogues	36	0 (0.0)	0 (0.0)	0 (0.0)	2 (5.6)	4 (11.1)	30 (83.3)	N/A	36 (100.0)
Plant-based cream analogues	22	0 (0.0)	0 (0.0)	7 (31.8)	1 (4.5)	12 (54.5)	2 (9.1)	0 (0.0)	15 (68.2)
Plant-based fat spread analogues	27	10 (37.0)	0 (0.0)	0 (0.0)	9 (33.3)	11 (40.7)	7 (25.9)	0 (0.0)	17 (63.0)
Plant-based ice cream analogues	23	0 (0.0)	0 (0.0)	0 (0.0)	4 (17.4)	18 (78.3)	1 (4.3)	N/A	23 (100.0)

^a^Agreement in classifying products as optimal nutritional quality options, i.e. eligible for Keyhole and qualified for Nutri-Score A or B

^b^Agreement in classifying products as suboptimal nutritional quality options, i.e. ineligible for Keyhole and qualified for Nutri-Score C, D or E

N/A (not applicable) is used when none of the products were assessed as high nutritional quality options by both FOPNL schemes

Plant-based cream analogues were ineligible for Keyhole; nevertheless, 32% of them qualified for Nutri-Score B. Agreement between these FOPNL schemes in suboptimal nutritional quality classification was observed in 15 out of 22 plant-based cream analogues. Although 37% of plant-based fat spread analogues were eligible for Keyhole, none qualified for Nutri-Score A or B, and thus, there was no agreement (0%) between two FOPNL schemes for classifying these analogues as optimal nutritional quality products. Plant-based ice cream analogues were ineligible for Keyhole and Nutri-Score A or B, and most of them qualified for Nutri-Score D (100% agreement between the FOPNL schemes in suboptimal nutritional quality classification).

Details of plant-based dairy analogues that did not meet the Keyhole criteria are shown in Appendix D. For the Keyhole assessable products, exceeding the total fat threshold was the main reason for the ineligibility of most plant-based milk, yoghurt, and cream analogues and the saturated fat to total fat ratio above the threshold was the main reason for the ineligibility of most plant-based fat spread analogues. In cheese analogues, exceeding the threshold for total fat and saturated fat to total fat ratio resulted in Keyhole ineligibility. The lack of compliance with Nutri-Score criteria was mainly due to low points for favourable components, as 71%, 99% and 96% of plant-based dairy analogues did not receive any points for protein, fibre and the percentage of fruit, vegetable and legumes, respectively. It is also worth noting that 83% of plant-based cheese analogues received the highest unfavourable point for the saturated fat component of the Nutri-Score algorithm.

Table 2 presents Keyhole eligibility and Nutri-Score categorisation of plant-based milk analogues based on their main ingredients. 29% of oat-based milk analogues were eligible for Keyhole; however, none qualified for Nutri-Score A or B. On the other hand, although none of the soy-, coconut-, pea- and mixed-based milk analogues were eligible for Keyhole, 93%, 100%, 80% and 44% qualified for Nutri-Score B, respectively. 12% of almond-based milk analogues were eligible for Keyhole, and 75% qualified for Nutri-Score B. In rice-based milk analogues, 60% were eligible for Keyhole, while only 20% qualified for Nutri-Score B. 33% of potato-based milk analogues were eligible for Keyhole and Nutri-Score B. Regardless of the main ingredient, there was no agreement between these two FOPNL schemes for classifying plant-based milk analogues as optimal nutritional quality products (except one product among rice-based milk analogues). The agreement for suboptimal nutritional quality classification varied from none in coconut- and broad bean-based milk analogues to 17 products in oat-based milk analogues.

**Table 2:** Nutritional quality of plant-based milk analogues according to their main ingredient based on Keyhole and Nutri-Score

	**n**	**Keyhole** **n (%)**	**Nutri-Score** **n (%)**					**Agreement in optimal nutritional quality**^a^ **n (%)**	**Agreement in suboptimal nutritional quality**^b^ **n (%)**
			**A**	**B**	**C**	**D**	**E**		
Oat	24	7 (29.2)	0 (0.0)	0 (0.0)	11 (45.8)	7 (29.2)	6 (25.0)	0 (0.0)	17 (70.8)
Soybean	15	0 (0.0)	0 (0.0)	14 (93.3)	1 (6.7)	0 (0.0)	0 (0.0)	0 (0.0)	1 (6.7)
Almond	8	1 (12.5)	0 (0.0)	6 (75.0)	2 (25.0)	0 (0.0)	0 (0.0)	0 (0.0)	1 (12.5)
Coconut	2	0 (0.0)	0 (0.0)	2 (100.0)	0 (0.0)	0 (0.0)	0 (0.0)	0 (0.0)	0 (0.0)
Rice	5	3 (60.0)	0 (0.0)	1 (20.0)	2 (40.0)	1 (20.0)	1 (20.0)	1 (20.0)	2 (40.0)
Pea	5	0 (0.0)	0 (0.0)	4 (80.0)	1 (20.0)	0 (0.0)	0 (0.0)	0 (0.0)	1 (20.0)
Potato	3	1 (33.3)	0 (0.0)	1 (33.3)	2 (66.7)	0 (0.0)	0 (0.0)	0 (0.0)	1 (33.3)
Cashew nut	1	0 (0.0)	0 (0.0)	0 (0.0)	1 (100.0)	0 (0.0)	0 (0.0)	N/A	1 (100.0)
Broad bean	1	0 (0.0)	0 (0.0)	1 (100.0)	0 (0.0)	0 (0.0)	0 (0.0)	0 (0.0)	0 (0.0)
Mixed^c^	9	0 (0.0)	0 (0.0)	4 (44.4)	4 (44.4)	0 (0.0)	1 (11.1)	0 (0.0)	5 (55.6)

^a^Agreement in classifying products as optimal nutritional quality options, i.e. eligible for Keyhole and qualified for Nutri-Score A or B

^b^Agreement in classifying products as suboptimal nutritional quality options, i.e. ineligible for Keyhole and qualified for Nutri-Score C, D or E

^c^Mixed-based products were soybean and pea, soybean and coconut, coconut and almond, and coconut and rice

N/A (not applicable) is used when none of the products were assessed as high nutritional quality options by both FOPNL schemes

Keyhole and Nutri-Score data for plant-based yoghurt analogues according to their main ingredient are displayed in Table 3. None of the plant-based yoghurt analogues were eligible for Keyhole except one mixed-based (oat and pea protein) product. 50% of oat-based and 100% of almond-based yoghurt analogues qualified for Nutri-Score B. 86% of soy-based and 63% of mixed-based yoghurt analogues were eligible for Nutri-Score A or B. All coconut-based yoghurt analogues qualified for Nutri-Score C. There was no agreement between FOPNL schemes for optimal nutritional quality classification of these analogues except for one mixed-based product. However, the agreement for suboptimal nutritional quality classification varied from none (almond-based) to all (coconut-based) products.

**Table 3:** Nutritional quality of plant-based yoghurt analogues according to their main ingredient based on Keyhole and Nutri-Score

	**n**	**Keyhole** **n (%)**	**Nutri-Score** **n (%)**					**Agreement in optimal nutritional quality**^a^ **n (%)**	**Agreement in suboptimal nutritional quality**^b^ **n (%)**
			**A**	**B**	**C**	**D**	**E**		
Oat	4	0 (0.0)	0 (0.0)	2 (50.0)	2 (50.0)	0 (0.0)	0 (0.0)	0 (0.0)	2 (50.0)
Soybean	21	0 (0.0)	7 (33.3)	11 (52.4)	3 (14.3)	0 (0.0)	0 (0.0)	0 (0.0)	3 (14.3)
Almond	3	0 (0.0)	0 (0.0)	3 (100.0)	0 (0.0)	0 (0.0)	0 (0.0)	0 (0.0)	0 (0.0)
Coconut	5	0 (0.0)	0 (0.0)	0 (0.0)	5 (100.0)	0 (0.0)	0 (0.0)	N/A	5 (100.0)
Mixed^c^	8	1 (12.5)	2 (25.0)	3 (37.5)	3 (37.5)	0 (0.0)	0 (0.0)	1 (12.5)	3 (37.5)

^a^Agreement in classifying products as optimal nutritional quality options, i.e. eligible for Keyhole and qualified for Nutri-Score A or B

^b^Agreement in classifying products as suboptimal nutritional quality options, i.e. ineligible for Keyhole and qualified for Nutri-Score C, D or E

^c^Mixed-based products were oat and pea protein, oat and potato protein, oat, coconut and broad bean protein, almond and potato protein

N/A (not applicable) is used when none of the products were assessed as high nutritional quality options by both FOPNL schemes

18% of organic plant-based milk analogues and none of the organic plant-based yoghurt analogues were eligible for Keyhole (Table 4). 16% and 3% of non-organic plant-based milk and yoghurt analogues were eligible for Keyhole, respectively. 64% of organic and 42% of non-organic plant-based milk analogues qualified for Nutri-Score B. In organic and non-organic plant-based yoghurt analogues, 50% and 74% of products qualified for Nutri-Score A or B, respectively. There was no agreement between these two FOPNL schemes for classifying organic plant-based milk and yoghurt analogues as optimal nutritional quality products, whereas the agreement for evaluating these analogues as suboptimal nutritional quality items was 18% and 50%, respectively.

**Table 4:** Nutritional quality of organic and non-organic plant-based milk and yoghurt analogues based on Keyhole and Nutri-Score

	**n**	**Keyhole** **n (%)**	**Nutri-Score** **n (%)**					**Agreement in optimal nutritional quality**^a^ **n (%)**	**Agreement in suboptimal nutritional quality**^b^ **n (%)**
			**A**	**B**	**C**	**D**	**E**		
*Plant-based milk analogues*									
Organic	11	2 (18.2)	0 (0.0)	7 (63.6)	3 (27.3)	0 (0.0)	1 (9.1)	0 (0.0)	2 (18.2)
Non-organic	62	10 (16.1)	0 (0.0)	26 (41.9)	21 (33.9)	8 (12.9)	7 (11.3)	1 (1.6)	27 (43.5)
*Plant-based yoghurt analogues*									
Organic	10	0 (0.0)	2 (20.0)	3 (30.0)	5 (50.0)	0 (0.0)	0 (0.0)	0 (0.0)	5 (50.0)
Non-organic	31	1 (3.2)	7 (22.6)	16 (51.6)	8 (25.8)	0 (0.0)	0 (0.0)	1 (3.2)	8 (25.8)

^a^Agreement in classifying products as optimal nutritional quality options, i.e. eligible for Keyhole and qualified for Nutri-Score A or B

^b^Agreement in classifying products as suboptimal nutritional quality options, i.e. ineligible for Keyhole and qualified for Nutri-Score C, D or E

The nutritional quality of plant-based cheese and cream analogues is shown in Table 5. None of the plant-based cheese analogues were eligible for Keyhole and Nutri-Score A or B, but most qualified for Nutri-Score E, regardless of whether they were plant fat-based or plant fat- and protein-based products. Thus, there was a complete agreement between these two FOPNL schemes in evaluating plant-based cheese analogues as suboptimal nutritional quality options despite their main ingredient. None of the plant-based cream analogues were eligible for Keyhole, but 38% and 17% of plant fat-based and plant fat- and protein-based products qualified for Nutri-Score B, respectively (no agreement between the FOPNL schemes for optimal nutritional quality classification). More than 56% of plant fat-based cream analogues qualified for Nutri-Score D or E, and 83% of plant fat- and protein-based cream analogues qualified for Nutri-Score D. Ten out of 16 and five out of six products in plant fat-based cream analogues and plant fat- and protein-based cream analogues were classified as suboptimal nutritional quality by both FOPNL schemes, respectively.

**Table 5:** Nutritional quality of plant-based cheese and cream analogues according to their main ingredient characteristics based on Keyhole and Nutri-Score

	**n**	**Keyhole** **n (%)**	**Nutri-Score** **n (%)**					**Agreement in optimal nutritional quality**^a^ **n (%)**	**Agreement in suboptimal nutritional quality**^b^ **n (%)**
			**A**	**B**	**C**	**D**	**E**		
*Plant-based cheese analogues*									
Plant fat-based cheese	21	0 (0.0)	0 (0.0)	0 (0.0)	0 (0.0)	2 (9.5)	19 (90.5)	N/A	21 (100.0)
Plant fat- and protein-based cheese^c^	15	0 (0.0)	0 (0.0)	0 (0.0)	2 (13.3)	2 (13.3)	11 (73.3)	N/A	15 (100.0)
*Plant-based cream analogues*									
Plant fat-based cream	16	0 (0.0)	0 (0.0)	6 (37.5)	1 (6.3)	7 (43.8)	2 (12.5)	0 (0.0)	10 (62.5)
Plant fat- and protein-based cream^d^	6	0 (0.0)	0 (0.0)	1 (16.7)	0 (0.0)	5 (83.3)	0 (0.0)	0 (0.0)	5 (83.3)

^a^Agreement in classifying products as optimal nutritional quality options, i.e. eligible for Keyhole and qualified for Nutri-Score A or B

^b^Agreement in classifying products as suboptimal nutritional quality options, i.e. ineligible for Keyhole and qualified for Nutri-Score C, D or E

^c^Protein-containing ingredients include nuts, seeds or protein isolates, e.g., potato protein

^d^Protein-containing ingredients include soybean or protein isolates, e.g., broad bean protein

N/A (not applicable) is used when none of the products were assessed as high nutritional quality options by both FOPNL schemes

**Fig. 1:**
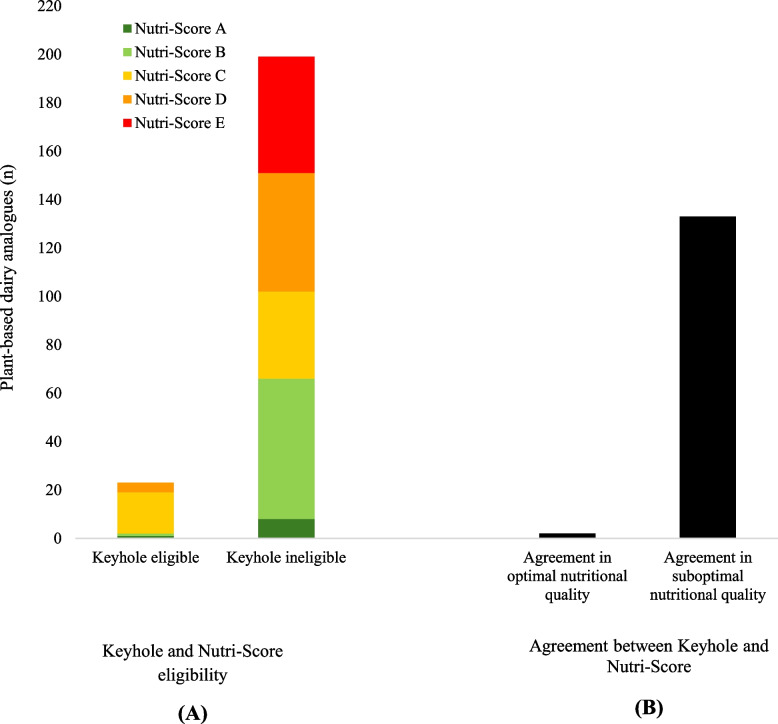
Nutritional quality of plant-based dairy analogues (*n* = 222) based on Keyhole and Nutri-Score assessment. (**A**) Keyhole and Nutri-Score eligibility of plant-based dairy analogues; (**B**) Agreement between Keyhole and Nutri-Score for nutritional quality assessment: the agreement for classifying the nutritional quality of products was defined as eligibility for Keyhole and Nutri-Score A or B (agreement in optimal nutritional quality) and ineligibility for Keyhole and qualifying for Nutri-Score C, D or E (agreement in suboptimal nutritional quality). The kappa value for the agreement between two FOPNL schemes is −0.13

The overall nutritional quality of plant-based dairy analogues according to Keyhole and Nutri-Score assessment is presented in Fig. 1. Of 222 plant-based dairy analogues, 23 (10%) and 68 (31%) products were eligible for Keyhole and Nutri-Score A or B, respectively. Keyhole and Nutri-Score had an agreement on classifying two plant-based dairy analogues as optimal nutritional quality products and 133 as suboptimal. The Kappa statistics indicated poor agreement between these two FOPNL schemes for the nutritional quality classification of plant-based dairy analogues (Kappa value = −0.13).

## Discussion

Consumers’ demand for plant-based foods is increasing, and introducing plant-based dairy analogues to the market is one of the strategies for addressing this demand. However, it is important to investigate the nutritional quality of these products and guide the public towards healthy choices. This study assessed the nutritional quality of 222 plant-based dairy analogues available in the Swedish market according to two main FOPNL schemes (Keyhole and Nutri-Score) in Europe.

The findings of this Swedish study demonstrated that the nutritional quality of each plant-based dairy analogue might be classified differently depending on the type of FOPNL scheme. Only two plant-based dairy analogues were classified as optimal nutritional quality products by both FOPNL schemes. Most Keyhole-eligible plant-based dairy analogues were plant-based fat spread analogues, whereas most Nutri-Score A or B-qualified products were plant-based yoghurt analogues. On the other hand, none of the plant-based cheese and ice cream analogues were classified as optimal nutritional quality products by Keyhole and Nutri-Score; thus, the agreement between these FOPNL schemes for suboptimal nutritional quality classification was 100% for these food groups. Moreover, a higher proportion of organic plant-based milk analogues were assessed as optimal nutritional quality products compared to non-organic varieties, while the opposite was true for organic plant-based yoghurt analogues.

Regarding the main ingredient, although oat-based milk analogues were dominant in terms of the number of products, none of them were classified as optimal nutritional quality analogues based on Nutri-Score, and a few were eligible for Keyhole. The dominant yoghurt analogues were soy-based products, the majority of which were classified as optimal nutritional quality options by the Nutri-Score but not the Keyhole. According to the findings of both FOPNL schemes, the suboptimal nutritional quality of plant-based cheese analogues did not vary despite differences in the main ingredient. However, Nutri-Score classified some plant fat-based cream analogues as optimal nutritional quality products even though the Keyhole assessment was contrary.

The findings of previous research on the application of Keyhole and the updated version of Nutri-Score to a small number of plant-based dairy analogues in Food Composition Databases were fairly in line with this Swedish market study. For example, plant-based yoghurt, cheese and cream analogues were ineligible for Keyhole, and the eligibility of plant-based fat spread analogues for this scheme varied [6]. In terms of Nutri-Score, most plant-based milk analogues qualified for Nutri-Score D and some for Nutri-Score B and C [9]. The plant-based yoghurt analogues had Nutri-Score A, B or C [6]. The plant-based cheese and cream analogues received Nutri-score D and B, respectively and the plant-based fat spread analogues qualified for Nutri-Scores C, D or E [6].

According to studies on the nutritional quality of plant-based milk analogues, these products were deemed optimal nutritional quality options as they qualified for Nutri-Score A or B [19, 21, 22]. However, based on the updated Nutri-Score algorithm (ineligibility of all beverages for Nutri-Score A, except water), none of the plant-based milk analogues of this Swedish study were eligible for Nutri-Score A, and less than 50% qualified for Nutri-Score B. Furthermore, this study showed that many of these products can have Nutri-Score C, D and E, particularly some of the oat- and rice-based milk analogues.

In the previous version of the Nutri-Score, the plant-based milk analogues were assessed according to the general food algorithm, while in the updated version, the beverage algorithm should be applied to these products [25]. In the Nutri-Score beverage algorithm, the threshold value for assigning points to unfavourable components such as energy and total sugar is lower than the general food algorithm [25]. Therefore, the application of the updated vs. previous algorithm results in a higher Nutri-Score for plant-based milk analogues. 

Reformulation is one of the strategies for complying with FOPNL schemes’ criteria. According to the findings of this Swedish study, the main reason for Keyhole ineligibility was the high fat content or high saturated fat to total fat ratio. The main reason for the high Nutri-Score was low protein, fibre, fruit, vegetable and legume content. Therefore, reformulating these analogues should focus on reducing the fat content and increasing favourable nutritional components. The reformulation of plant-based dairy analogues is complicated, and some issues faced by the food industry include unpleasant sensory characteristics, stability, shortcomings in nutrient profile and the presence of antinutrients [28–30]. The reformulation can be optimised from the nutritional perspective by nutrient fortification or selecting certain ingredients to address nutrient inadequacies and minimise antinutrient impact.

There are some challenges in considering FOPNL as an indicator of nutritional quality for plant-based dairy analogues. In this Swedish study, most plant-based dairy analogues identified as optimal nutritional quality products by Nutri-Score were ineligible for Keyhole. Thus, these FOPNL schemes mostly disagreed on classifying the plant-based dairy analogues as having optimal nutritional quality; however, their agreement was higher when classifying them as having suboptimal nutritional quality. Since the criteria for Keyhole eligibility differs from Nutri-Score, applying these two FOPNL schemes for nutritional quality evaluation may lead to contradictory results and can be confusing for the consumers if both are used simultaneously. A previous comparison of these two FOPNL schemes for nutritional assessment of different foods in the Swedish Food Composition Database showed an overall good agreement; however, plant-based milk analogues were excluded, and other plant-based dairy analogues were not assessed as a separate food group and in detail [6].

FOPNL schemes are designed to help consumers select healthy foods and encourage manufacturers to develop or reformulate products with healthier profiles. In the case of plant-based dairy analogues, Keyhole and Nutri-Score partially fulfil these goals by differentiating products with lower fat, sugar, and salt, as well as higher protein and fibre content. However, they cannot distinguish between fortified and unfortified products, as seen in the comparison of organic and non-organic plant-based milk and yoghurt analogues. The micronutrient fortification of these innovative analogues is essential to make them comparable to dairy products [31]. Currently, the micronutrient content of foods (naturally rich or fortified) does not affect the assessment of products by these FOPNL schemes. This is one of the limitations of Keyhole and Nutri-Score applications for the nutritional assessment of foods that require fortification, such as plant-based dairy analogues. Therefore, integrating the micronutrient content of foods in FOPNL schemes to guide consumers towards nutritious plant-based dairy analogues warrants further consideration.

Comparing the nutritional profiles of plant-based dairy analogues to traditional dairy reveals some differences in their nutritional quality [18, 32]. However, due to the limitations of Keyhole and Nutri-Score, such as excluding micronutrients in the assessment, these differences may not appear in comparing traditional dairy and plant-based dairy analogues, and the role of fortification fades in this context. For example, plain milk is ineligible for Keyhole unless it contains 0.7 g/100 g fat or less [1], so most plain and all flavoured milk are ineligible for the Keyhole. This Swedish study found several keyhole-eligible plant-based milk analogues in the market. Regarding Nutri-Score, skim and semi-skim milk generally qualify for Nutri-Score B, while whole milk mostly qualifies for Nutri-Score C [25]. The Nutri-Score range for the plant-based milk analogues in this study was more diverse than that of milk.

The comparison of the nutritional quality of other dairy products from Food Composition Databases with their plant-based analogues in this Swedish study highlights similar shortcomings in these FOPNL schemes. For example, only some plain and flavoured low-fat yoghurts are eligible for Keyhole, but the Nutri-Score of different types of yoghurt ranges from A to C [6]. The nutritional quality of plant-based yoghurt analogues in this Swedish study was similar to that of yoghurt. Some cheeses with 17 g/100 g fat or less are Keyhole eligible [1, 6]. The Nutri-Score of cheeses also varies between B and E [6]; thus, this group has some optimal nutritional quality choices compared to plant-based cheese analogues. Cream, butter and ice cream are ineligible for Keyhole, but some margarines are eligible [6], and all are considered suboptimal nutritional quality items in Nutri-Score assessment [6, 9]. The findings of this study for plant-based analogues of these foods were similar except for Nutri-Score of cream analogues. The difference in nutritional quality assessment of the dairy products and their plant-based analogues by Keyhole and Nutri-Score is independent of their vitamin and mineral content.

In addition to Keyhole and Nutri-Score, some studies have used other methods to assess the nutritional quality of plant-based dairy analogues. For example, Drewnowski [21] proposed using Nutrient Standards for the transparent labelling of plant-based dairy analogues. In this method, the nutrient content of plant-based milk analogues was assessed in comparison with milk and based on nutrition recommendations (percentage of Daily Value) for energy and some nutrients such as protein, sugar, saturated fat, sodium, calcium, vitamin B2 and B12 [21, 33]. Other studies have also used similar methods by defining some criteria according to dietary guidelines and the percentage of Daily Values for plant-based milk, yoghurt and cheese analogues [34–36].

Another method for nutritional quality assessment of these plant-based dairy analogues is the Nutrient Rich Food (NRF) index, which may be more useful for differentiating foods based on their micronutrient content compared to Keyhole and Nutri-Score. NRF index evaluates the nutritional quality of products based on nutrients to encourage and limit [37, 38]. NRF 5.3 has been used for plant-based milk analogues by considering protein, vitamins A, D, B12 and calcium as five nutrients to encourage and saturated fat, total sugar and sodium as three nutrients to limit [21].

Keyhole and Nutri-Score calculations have some limitations. As mentioned, Keyhole eligibility is limited to specific groups; for example, flavoured plant-based milk analogues were not considered for the Keyhole eligibility assessment. Thus, all flavoured varieties of these beverages are deemed Keyhole ineligible regardless of their nutrient content. The limitation of Nutri-Score calculation was the lack of data on the fibre content of some plant-based dairy analogues, which may affect the Nutri-Score value. However, in the Nutri-Score algorithms, the fibre threshold for gaining a point was 3 g per 100 g, which is less likely to be the case for these analogues as seen for similar products [18]. Furthermore, earning extra points does not necessarily result in a change in the Nutri-Score category. Thus, this limitation may not significantly affect our findings. Similarly, the lack of declaration of fruit, vegetable and legume percentages in some of the products’ ingredient lists was another limitation of the Nutri-Score calculation. Nevertheless, since the threshold for obtaining a point for this component was 40%, it is less likely to affect our results.

In addition to practical issues in Keyhole and Nutri-Score calculations, this study had some strengths and limitations. This was the first study to use Keyhole and the updated version of the Nutri-Score algorithm to assess the nutritional quality of plant-based dairy analogues in the Swedish market. However, the findings may not be generalisable to other markets due to the diversity of ingredients in these innovative products. Various plant-based dairy analogue groups were evaluated in this study, but not all products in the market were covered. Moreover, the eligibility for the Keyhole and Nutri-Score categories was assessed based on the manufacturer’s declaration of nutrient content on the product package or website instead of laboratory analytical data. Future studies should focus on a broader range of plant-based dairy analogues, particularly new emerging products in the dynamic market, and use analytical and recipe data for more accurate calculations.

## Conclusion

The results of this study highlight significant variability in the eligibility of plant-based dairy analogues for Keyhole and Nutri-Score labelling. Eligibility for Keyhole was highest among plant-based fat spread analogues, while Nutri-Score A and B ratings were more common for plant-based yoghurt analogues. Plant-based cheese and ice cream analogues were ineligible for both FOPNL schemes. The nutritional quality of plant-based dairy analogues may vary, even within product categories based on the same main ingredient. Furthermore, Keyhole and Nutri-Score assessment criteria do not address concerns regarding the micronutrient content of these analogues; therefore, the micronutrient content of organic and non-organic plant-based dairy analogues did not affect their evaluation by Keyhole and Nutri-Score.

The assessment of plant-based dairy analogues based on Keyhole and Nutri-Score criteria demonstrated that the interpretation of the nutritional quality within the food category might depend on the type of FOPNL scheme. The Keyhole and Nutri-Score often diverged in classifying most plant-based dairy analogues as optimal nutritional quality products; however, they showed greater alignment in categorising these analogues as suboptimal nutritional quality products, highlighting differences in their assessment criteria and potential implications for consumer guidance. These results emphasise the need for comprehensive assessments and complementary labelling systems to effectively guide consumer choices across diverse plant-based dairy analogues. Meanwhile, the limitations of these FOPNL schemes should be taken into consideration when evaluating the nutritional quality of plant-based dairy analogues.

## Supplementary Information

Supplementary Material 1.

## Data Availability

The datasets used during the current study are available from the corresponding author upon reasonable request.
