# Supplementary material for: Application of Nordic Keyhole and Nutri-Score for assessment of nutritional quality of plant-based dairy analogues
Source: BMC Nutr. 2025 Feb 28;11:45. doi: 10.1186/s40795-025-01023-3 (PMC11869733; doi:10.1186/s40795-025-01023-3)
Supplement: Supplementary file 1 — Supplementary Material 1. [file 40795_2025_1023_MOESM1_ESM.pdf]

Appendix A. Keyhole threshold for plant-based dairy analogues[1]

	Total fat/100g	Saturated to total fat ratio	Total sugar/100 g	Salt/100 g
Plain plant-based milk	≤ 1.5 g	≤ 33 %	≤ 5 g	≤ 0.1 g
Plain and flavoured plant-based yoghurt	≤ 1.5 g	≤ 33 %	P: ≤ 5 g, F: ≤ 8 g	≤ 0.1 g
Plain and flavoured plant-based cheese	≤ 17 g	≤20 %	-	≤ 1.5 g
Plain and flavoured plant-based cream	≤ 5 g	≤ 33 %	≤ 5 g	P: ≤ 0.3 g , F: ≤ 0.8 g
Plant-based fat spread	≤ 80 g	≤33 %	-	≤ 1.1 g
Plant-based liquid fat spread	-	≤20 %	-	≤ 1g
Plant-based ice cream	N/A	N/A	N/A	N/A

[1] Threshold values adopted from the Swedish Food Agency Keyhole regulation

P: Plain

F: Flavoured

N/A: Not applicable

Appendix B. Nutri-Score classification for plant-based dairy analogues based on Nutri-Score algorithm 2023[1]

	Plant-based milk analogues	Plant-based fat spread and cream analogues	Other plant-based dairy analogues
Nutri-Score A (dark green)	N/A	≤ -6	≤ 0
Nutri-Score B (green)	≤ 2	-5 to 2	1 to 2
Nutri-Score C (Yellow)	3 to 6	3 to 10	3 to 10
Nutri-Score D (orange)	7 to 9	11 to 18	11 to 18
Nutri-Score E (dark orange)	≥ 10	≥ 19	≥ 19

[1] Values adopted from the Public Health Agency of France Nutri-Score regulation

Appendix C. Nutritional quality of plant-based dairy analogues based on Keyhole and Nutri-Score

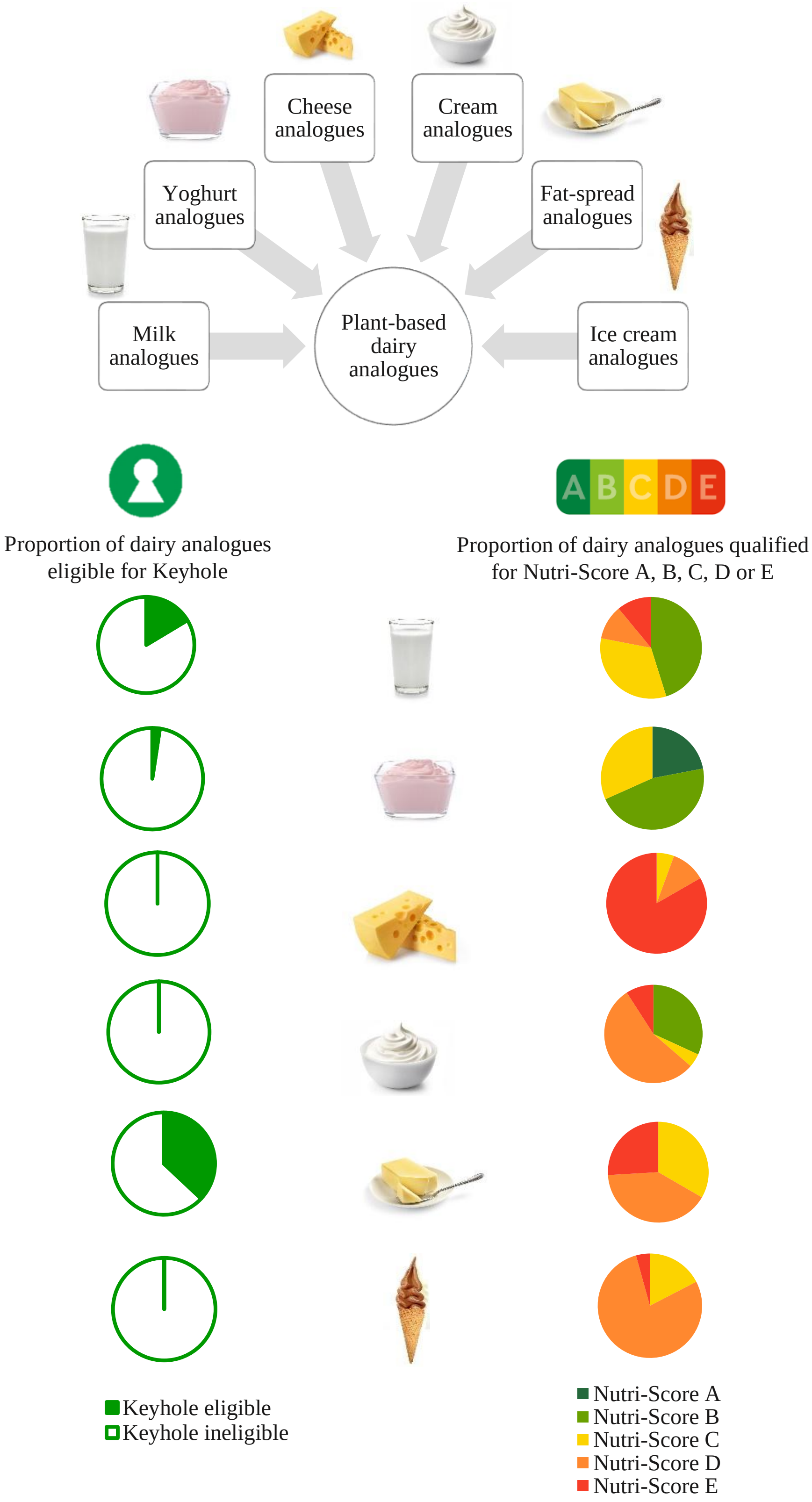

Appendix D. Number of plant-based dairy analogues not meeting the threshold for the Keyhole criteria

	Assessable products (n)[1]	Total fat (n)	Saturated to total fat ratio (n)	Total sugar (n)	Salt (n)
Plant-based milk analogues	59	25	13	5	19
Plant-based yoghurt analogues	41	38	8	12	20
Plant-based cheese analogues	27	25	27	N/A	24
Plant-based cream analogues	22	22	15	2	3
Plant-based fat spread analogues	25	3	10	N/A	8
Plant-based ice cream analogues	0	N/A	N/A	N/A	N/A

[1]Plant-based dairy analogues eligible for assessment against Keyhole criteria

N/A: not applicable
